# Eczema and related atopic diseases are associated with increased symptom severity in children with autism spectrum disorder

**DOI:** 10.1038/s41398-022-02185-5

**Published:** 2022-09-28

**Authors:** C. Jameson, K. A. Boulton, N. Silove, A. J. Guastella

**Affiliations:** 1 Clinic for Autism and Neurodevelopment (CAN) Research, Brain and Mind Centre, Children’s Hospital Westmead Clinical School, Faculty of Medicine and Health, University of Sydney, Camperdown, Australia; 2 Child Neurodevelopment and Mental Health Team, Brain and Mind Centre, University of Sydney, Camperdown, Australia; 3 Child Development Unit, The Children’s Hospital at Westmead, Westmead, NSW 2145 Australia

**Keywords:** Predictive markers, Physiology

## Abstract

Growing evidence indicates that autism spectrum disorder (ASD) has diverse genetic, neurological, and environmental factors that contribute to its neurodevelopmental course. Interestingly, childhood ASD is often accompanied by skin disorders, such as eczema, and other related atopic manifestations. This link may be due to the shared embryonic origin of epidermal and neural tissue. Accordingly, we consider the potential influence of a skin-brain co-vulnerability and ensuing atopic cascade on ASD symptomatology by investigating whether atopic disorders (asthma, allergies, eczema and hay fever) are associated with increased symptom severity in children with ASD. Overall, 45 atopic and 93 non-atopic children with ASD were assessed using the ADOS-2 on scores of total, social and non-social symptoms. Differences in ASD symptom severity were further evaluated as a function of atopic disease type. Atopic children displayed greater symptom severity overall and in the social domain, relative to non-atopic participants. Atopic children were 2.4 times more likely to experience overall impairments classified within the ADOS-2 highest-level severity bracket and 2.7 times more likely to show social difficulties in this range. Moreover, those reporting comorbid eczema displayed increased symptom severity relative to both their non-atopic peers and those reporting asthma and allergies. Taken together, findings indicate that atopic disorders, and particularly comorbid eczema, are associated with increases in ASD symptom severity. Findings provide grounds for future investigations into this link between childhood skin diseases and ASD symptom severity to advance our understanding of neurodevelopment and to develop targeted assessment and intervention opportunities.

## Introduction

Autism spectrum disorders (ASD) represent a group of neurodevelopmental conditions characterised by reduced social responsivity and restricted and stereotyped interests and behaviours [1]. The aetiology of ASD is underpinned by a complex interplay between neurological, genetic, and environmental factors that culminate in heterogeneous clinical presentations [2]. Consequently, ASD is increasingly recognised as representing a broad range of neurodevelopmental disorders and subtypes that are associated with diverse physical and mental health comorbidities [3].

The role of atopic diseases in neurodevelopment has received growing attention as a potential modifier of neurodevelopment [4–6]. Although atopic disorders are relatively common, affecting between 5 and 15% of all children, accumulating evidence indicates prevalence of such diseases is increased amongst those with ASD [6, 7]. For instance, in a recent meta-analysis including over one million pooled participants, Tasi et al. [8] determined that children with ASD were 1.49 times more likely to experience atopic dermatitis relative to their typically developing peers (95% CI: 1.2–1.8). Similarly, Magalhães et al. [9] reported that 86.6% of participants with Asperger’s syndrome (*N* = 15) had at least one atopic disease, compared to <7% in a neurotypical cohort. Further, children with ASD appear 4.5 times more likely to report food allergy (95% CI: 3.0–7.0) [7], and experience significantly increased rates of asthma and allergic rhinitis relative to those without ASD (OR 1.69, 95% CI: 1.11–2.59 and OR 1.66, 95% CI: 1.49–1.85 respectively) [10]. Notably, these findings have been reported across large and multi-racial samples, suggesting this link extends beyond ethnic or cultural differences. Overall, whilst a positive association between atopic manifestations and ASD has been repeatedly observed, this association with neurodevelopmental outcomes remains poorly understood.

Research examining whether atopic disorders are associated with the severity of neurodevelopmental delay in ASD is scarce. In the only study to date, Mostafa, Hamza and El-Shahawi (2008) evaluated reports of atopic disease (bronchial asthma, atopic dermatitis, and allergic rhinitis) amongst children diagnosed with autism (*N* = 50) relative to an age- and sex-matched neurotypical cohort (*N* = 50) [11]. The researchers observed that children with ASD who scored in the severe range on the Childhood Autism Rating Scale (CARS) (*N* = 20) showed significantly increased rates of atopic disease (80%), compared to both children without an autism diagnosis (10%) and those with mild to moderate autism presentations (33%). The authors, however, did not investigate symptom domains associated with ASD, nor did they distinguish between cutaneous and non-cutaneous atopic disorders, despite growing recognition of a skin-brain axis stemming from a shared embryonic origin [12, 13].

The skin-brain axis is of emerging interest in the field of neurodevelopment and has prompted discussion as to whether atopic eczema’s apparent link with ASD may be unique from that of non-cutaneous atopic conditions. For instance, from 3 to 4 post-conception weeks, the brain, skin, and skin appendages develop in synchronisation in utero, all originating from one of three primary germ layers, the ectoderm [12]. Accordingly, whilst the neuroectoderm differentiates to form early derivatives of the brain, such as the neural tube, the ventral ectoderm develops into a monolayer epidermis [13]. It, therefore, seems plausible that atopic eczema may be uniquely associated with neurodevelopmental outcomes relative to non-cutaneous atopic disorders. Interestingly, cutaneous syndromes also often mark the onset of the “atopic march”, with epidermal dysfunction facilitating subsequent allergic sensitisation and atopic respiratory disorders [14]. Accordingly, in eczematic patients, sub-optimal tight junctions in the epidermis may be accompanied by disrupted barrier function in the airway epithelium or intestinal mucosa, easing the transcutaneous invasion of allergens and inducing a maladaptive immune response [15]. Notably, this systemic and hyper-sensitive atopic response has been observed to reduce the integrity of the blood-brain-barrier, facilitating excess cytokine entry into the brain and potentiating neuroinflammation [16–18]. Accordingly, previous studies have reported positive correlations between elevated plasma and serum concentrations of atopic inflammatory markers, including interleukin (IL)-4 and IL-17A, and increasingly pronounced behavioural impairments in children with ASD [19]. Therefore, despite acknowledging a likely skin-brain connection and appreciating the influence of atopic barrier dysfunction on neurodevelopment, it remains unclear whether atopic eczema may influence neurodevelopment in a manner distinct from related respiratory and gastrointestinal atopies.

In this study, we explored whether atopic comorbidities (asthma, allergies, eczema and hay fever) were associated with increased symptom severity in a cohort of children with ASD. Specifically, we aimed to determine whether symptom severity differed between children with ASD, with and without atopic complaints, including overall symptoms as well as social affect (SA) and restricted and repetitive behaviours (RRB). Additionally, we aimed to identify whether reporting an atopic condition increased a child’s risk of displaying symptoms classified within the severe range and determined whether ASD-related behavioural impairments varied as a function of atopic disease type for each domain. We hypothesised that, relative to those without an atopic complaint, children with ASD and an atopic comorbidity would present with increased ASD symptomatology overall and across both SA and RRB domains. Further, we proposed that atopic children would be more likely to display symptoms classified as severe, compared to children without an atopic comorbidity. Additionally, given the proposed role of cutaneous syndromes as precursors to related atopic conditions, we hypothesised that children with comorbid eczema would show increased symptom severity in each of these domains relative to both non-atopic children and those reporting other prevalent atopic conditions.

## Method

### Participant recruitment

Participants included children with ASD who were recruited from the Clinic for Autism and Neurodevelopment (CAN) Research, located at the Brain and Mind Centre, University of Sydney (*n* = 105) and the Child Development Unit, located in Westmead’s Children’s Hospital (*n* = 35). Overall, 140 children were included in the study (*n* = 110 males, 30 females; mean age 6.14 [standard deviation = 2.62]). All participants were ambulatory with no suspected vision or hearing impairments. The Sydney Children’s Hospitals Network (SCHN) Human Research Ethics Committee (HREC) (LNR/17/SCHN/293), University of Sydney HREC (2013/502) or Sydney Local Health District (SLHD) HREC (18/RPAH/157) provided approval. Informed consent was obtained from all parents or caregivers before participation.

A diagnosis of ASD was confirmed using the Autism Diagnostic Observation Schedule, Second Edition (ADOS^®^-2) [20]. All participants were assessed using either ADOS-2 Module 1 (for children with few-to-no words, *n* = 70), Module 2 (for those with phrase speech, *n* = 40) or Module 3 (for verbally fluent children and young adolescents, *n* = 28). The presence of any auto-immune, neurological (e.g., seizures) or medical conditions were also considered exclusion criteria for all participants [6, 19]. Six children were excluded due to reports of systemic lupus erythematosus (*n* = 1), an unspecified thyroid disease (*n* = 1), Klinefelter syndrome (*n* = 1), Phelan–McDermid syndrome (*n* = 1), Prader–Willi syndrome (*n* = 1) and a seizure disorder (*n* = 1).

### Developmental history questionnaire

All participants were screened using a self-reported measure for atopic diseases, electronically completed by the child’s parent or caregiver prior to attending their appointment. Atopic conditions included asthma, allergies, eczema and hay fever. The participant’s medical history data, including the presence of comorbid auto-immune and mental health conditions, and atopic symptom onset, were evaluated via the questionnaire. Participants’ sleep and maternal immune histories were additionally collected. Medical diagnoses were corroborated in an unstructured medical interview.

Participants were subsequently classified into two groups: an atopic cohort (one or more of the above atopic conditions) and a non-atopic cohort. In subsequent analyses, the atopic cohort was further stratified by atopic disease type, matched on age and sex where possible, enabling direct comparisons between eczematic participants and children reporting either asthma/allergies or no atopic condition. Parent/caregiver responses were collected and managed using REDCap (Research Electronic Data Capture) hosted at the University of Sydney [21, 22].

### ASD symptom severity

ASD severity was measured using ADOS calibrated severity scores (CSSs) calculated for the subdomains of social affect (CSS-SA) and restricted and repetitive behaviour (CSS-RRB), as well as an overall CSS, where 1 represents minimal-to-no evidence of ASD-related symptoms and 10 indicates a high degree of impairment. Participant CSS scores were computed using existing validated algorithms [20, 23]. As recommended by prior literature [24, 25], CSS scores were used instead of raw scores to directly compare symptom severity across different ADOS modules.

### Statistical analyses

Shapiro–Wilk normality tests indicated that several variables were non-normally distributed, as shown in Supplementary Table 1. Consequently, all analyses were conducted using bootstrapping (5000 resamples), enabling parametric statistical procedures to be applied. Demographic characteristics of children in the atopic and non-atopic cohort, as well as children reporting eczema relative to other atopic conditions, were compared using Welch two independent-sample *t*-tests and *χ*^2^ tests, as appropriate. Additionally, Fisher’s exact tests were used to compare sleep behaviours between the atopic and non-atopic groups due to the reduced sample of available data, with sleep history only collected at one study site.

Differences in mean ADOS CSS-SA, CSS-RRB and CSS scores for children in the atopic and non-atopic cohort were determined using Welch two independent-sample *t*-tests. The distribution of participants’ ADOS scores, including mean ± standard deviation (SD), were subsequently graphed using a truncated violin plot. The same statistical procedures were applied to determine differences in mean ADOS CSS-SA, CSS-RRB and CSS scores for children with comorbid eczema relative to age- and sex-matched non-atopic and asthma/allergies cohorts. Additionally, binomial logistic regressions were used to investigate whether ≥1 atopic comorbidity increased the likelihood of a participant presenting with ASD symptomatology exclusively defined within the severe range (score ≥7 on the CSS-SA, CSS-RRB and overall CSS scales), generating odds ratios (ORs) and 95% confidence intervals (CIs). Participants with missing data for a particular outcome were excluded from the analysis for that measure.

Statistical analyses were performed using R for macOS 10.13, version 4.1.0 (RStudio, Boston) and graphs were created using GraphPad Prism version 9.0.0 for macOS (GraphPad Software, California). A two-sided value of *p* < 0.05 was considered statistically significant. Sample size was based on a sample of convenience but was powered to a similar degree to past studies examining related variables [26].

## Results

### Participant characteristics

Table 1 presents the demographic characteristics of the sample. Of the total 140 children with ASD included in the study, atopic conditions of any type were found in 47 (33.57%) participants (atopic cohort, *n* = 39 males, 8 females; mean age 6.13 [standard deviation = 2.69). The remaining 93 (66.43%) participants did not report any atopic conditions, serving as a comparison group (non-atopic cohort, *n* = 71 males, 22 females; mean age 6.14 [standard deviation = 2.61]). There were no statistically significant differences between the atopic and non-atopic cohorts in sex, age, or reports of maternal infection (*p* > 0.05). Additionally, of the 66 participants with available sleep disturbance data (*n* = 17 atopic, *n* = 49 non-atopic), no significant differences were detected between the atopic and non-atopic groups in the occurrence of any undesirable sleep behaviours.

**Table 1:** Demographic characteristics of children with ASD in the atopic and non-atopic cohorts.

	Full cohort^a^	Atopic cohort	Non-atopic cohort	*p* value
Number of participants, *n* (%)	140 (100)	47 (33.57)	93 (66.43)	–
Asthma	15 (10.70)	–	–	–
Allergies	23 (16.40)	–	–	–
Eczema	26 (18.60)	–	–	–
Hay fever	2 (1.40)	–	–	–
Sex, *n* (%)				0.366^b^
Male	110 (78.60)	39 (82.98)	71 (76.34)	–
Female	30 (21.40)	8 (17.02)	22 (23.66)	–
Age (years), mean ± SD	6.15 ± 2.62	6.13 ± 2.69	6.14 ± 2.61	0.983^c^
Sleep behaviours, *n* (%)				
Poor sleeping pattern	22 (33.85)	6 (35.30)	16 (33.33)	0.883^d^
Snoring	10 (15.38)	4 (23.53)	6 (12.50)	0.434^d^
Trouble falling asleep	13 (20.00)	4 (23.53)	9 (18.75)	0.729^d^
Dislikes sleeping alone	15 (23.08)	6 (35.30)	9 (18.75)	0.191^d^
Waking overnight	17 (26.15)	3 (17.65)	14 (29.17)	0.523^d^
Maternal infection, *n* (%)	21 (15.22)	8 (17.02)	13 (13.98)	0.560^b^

*SD* standard deviation.

^a^Percentage of missing data in each measure for full cohort: maternal infection 1.43%. Sleep behaviours 47.14%. Percentages are expressed as a fraction of the number of participants in each group and may not total 100 due to missing data for some variables. *p* values are reported for comparisons between the atopic and non-atopic cohort using the *χ*^2^ test.

^b^Bootstrapped Welch two-sample *t*-test.

^c^Or Fisher’s exact tests.

^d^As appropriate.

### Scale scores and assessments of normality

Supplementary Table 1 shows descriptive data for the sample, including scale scores and assessments of normality. The Shapiro–Wilk normality test on all participants revealed a non-normal distribution for age, with the variable showing a positive skew towards early childhood. Similarly, ADOS-2 CSS total, CSS-SA and CSS-RRB scores showed non-parametric distributions, with a negative skew towards scores indicative of a high level of impairment.

### ASD phenotypic outcomes

**Fig. 1:**
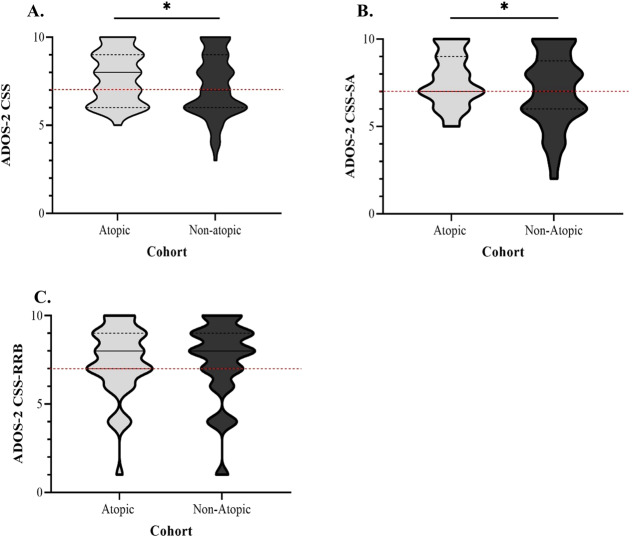
Distribution of ADOS scores in the atopic (*n* = 47) and non-atopic cohorts (*n* = 93). Children with an atopic comorbidity scored significantly higher on the **A**. ADOS-2 CSS (*p* = 0.039) and **B**. CSS-SA (*p* = 0.046) relative to those without an atopic disorder. No significant differences were detected in **C**. CSS-RRB scores between the atopic and non-atopic cohorts (*p* = 0.943). All *p* values are reported for a difference in mean between the atopic and non-atopic cohort, as determined using bootstrapped Welch two independent-sample *t*-tests. *p < 0.05. The black solid lines represent the median and the black dotted lines represent the quartiles. The horizontal red dotted line represents the threshold for severe ASD-related symptomatology of the ADOS (CSS = 7 or above). The width of the curves corresponds to the frequency of each ADOS score. Percentage missing data for full cohort: ADOS-2 all scales 3.6%.

ASD symptom severity was compared between the atopic and non-atopic cohort, as indexed by ADOS CSS total and domain-specific scores. Overall, children presenting with an atopic comorbidity showed significantly higher mean ADOS CSS scores on the total scale (7.79 ± 1.51) compared to those in the non-atopic cohort (7.16 ± 1.86; *t* = −2.09, df = 99.85, *p* = 0.039), as illustrated in Fig. 1A. Similarly, Fig. 1B indicates that children with an atopic disease recorded significantly higher mean scores on the ADOS CSS-SA subscale (7.63 ± 1.51) relative to children without an atopic comorbidity (7.00 ± 2.07; *t* = −1.99, df = 109.15, *p* = 0.046). In contrast, considering the effect of atopic conditions on ADOS CSS mean scores in the RRB symptom domain, no significant difference was observed between children in the atopic (7.42 ± 1.94) and non-atopic cohorts (7.45 ± 2.22; *t* = 0.07, df = 92.78, *p* = 0.943), as shown in Fig. 1C.

Given the statistically significant differences between the atopic and non-atopic groups in ADOS CSS total and CSS-SA scores, subsequent analyses were conducted to evaluate whether reporting an atopic condition increased a child’s likelihood of showing symptomatology classified in the severe range. This was indexed by an ADOS score of ≥7 on total and domain-specific subscales, indicative of the highest level of impairment. In these analyses, atopic children were 2.3 times more likely to receive a score in the upper threshold on the ADOS CSS total scale relative to non-atopic children (CI: 1.01–5.04, *p* = 0.047), as shown in Table 2. Similarly, children with an atopic condition were 2.9 times more likely than non-atopic participants to score in the severe range on the CSS-SA subscale (CI: 1.26–6.84, *p* = 0.013).

**Table 2:** Generalised linear models evaluating the presence of at least one atopic condition as a predictor of moderately high to severe ASD-related symptomatology in children.

	*β*	SE	*z*-value	OR	95% CI	*P* (Wald’s test)
ADOS CSS ≥7	0.81	0.41	1.98	2.25	1.01–5.04	0.047^*^
ADOS CSS-SA ≥7	1.08	0.43	2.49	2.93	1.26–6.84	0.013^*^
ADOS CSS-RRB ≥7	0.17	0.45	0.38	1.19	0.49–2.86	0.704

*CSS* calibrated severity scores, *SA* social affect, *RRB* restricted and repetitive behaviour.

ADOS-2 autism diagnostic observation schedule, second edition; CSS, SA, RRB. Percentage of missing data in each measure for full cohort: maternal infection 1.45%, ADOS-2 all scales 3.6%. *p *<* 0.05.

### ASD symptom severity as a function of disease type

We then looked at differences in ADOS CSS and CSS-SA scores as a function of atopic disease type. In these analyses, the full cohort was stratified into three subgroups: eczematic (*n* = 26), non-atopic (*n* = 24) and asthma/allergies (*n* = 17), individually matched on age and sex where possible. Table 3 presents the demographic characteristics for each of these cohorts. There were no significant differences between the eczema and non-atopic or asthma/allergies groups in terms of sex (*p* = 0.769 and *p* = 0.844 respectively) or age (*p* = 0. 971 and *p* = 0.557).

**Table 3:** Demographic characteristics of children with ASD and comorbid eczema, non-atopic or comorbid asthma/allergies (*n* = 67).

	Eczema cohort	Non-atopic cohort	Asthma/allergies cohort
Number of participants	26	24	17
Sex, *n* (%)			
Male	22 (84.6)	21 (87.5)	14 (82.4)
Female	4 (15.4)	3 (12.5)	3 (17.6)
Age (years), mean ± SD	6.10 ± 2.84	6.07 ± 2.85	5.64 ± 2.30

*SD* standard deviation.

**Fig. 2:**
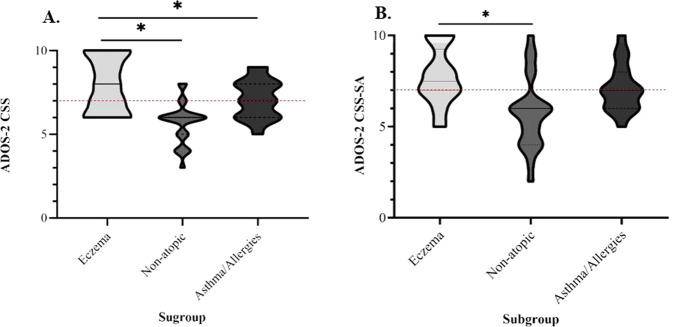
Distribution of ADOS scores in the eczematic (*n* = 26), non-atopic (*n* = 24) and asthma/allergies (*n* = 17) subgroups. Children with comorbid eczema scored significantly higher on the **A**. ADOS-2 CSS relative to those in the non-atopic subgroup (*p* < 0.001), and those in the asthma allergies group (*p* = 0.034 using one-tailed test; *p* = 0.069 using two-tailed test). **B** Children with comorbid eczema scored significantly higher on the ADOS-2 CSS-SA relative to those in the non-atopic subgroup (*p* = 0.001) yet no differences were observed relative to the asthma/allergies cohort (*p* = 0.197). All *p* values were determined using bootstrapped Welch two independent-sample *t*-tests. *p < 0.05. The black solid lines represent the median and the black dotted lines represent the quartiles. The horizontal red dotted line represents the threshold for severe ASD-related symptomatology of the ADOS (CSS = 7 or above). The width of the curves corresponds to the frequency of each ADOS score.

As expected, children with ASD and comorbid eczema received significantly higher mean scores on the ADOS CSS total scale (7.95 ± 1.62) relative to those in the non-atopic cohort (5.71 ± 1.27; *t* = 5.35, df = 39.29, *p* < 0.001). The comparison between the eczema and asthma/allergies subgroups was only significant in a one-tailed test (*p* = 0.034; two-tailed test: *t* = 1.88, *df* = 36.86, *p* = 0.069), as shown in Fig. 2A. As depicted in Fig. 2B, children with ASD and comorbid eczema also displayed significantly higher mean scores on the ADOS CSS-SA (7.73 ± 1.58) compared to those in the non-atopic cohort (5.75 ± 1.96; *t* = 3.78, df = 43.31, *p* = 0.001), yet not those in the asthma/allergies cohort (7.12 ± 1.32; *p* = 0.197).

## Discussion

To our knowledge, this is the first study to examine whether children with ASD and atopic conditions display increased autism-related symptom severity, with a particular focus on comorbid eczema. Findings showed that children with ASD and atopic disease experienced more pronounced symptoms overall and on the ADOS social domain, relative to those children with ASD without atopic complaints. That is, relative to the non-atopic cohort, atopic children were 2.3 times more likely to experience overall symptoms classified within the ADOS-2 highest-level severity bracket and 2.9 times more likely to show social difficulties in this range. Furthermore, the current findings suggested that ASD symptom severity may vary as a function of atopic disease type, with eczematic children displaying a greater degree of overall impairment relative to both age- and sex-matched non-atopic participants and those reporting other prevalent atopies (asthma/allergies). These findings support further investigations of a possible early life link between cortical and epidermal development, underpinned by a possible skin-brain-immune connection. This research may lead to the identification of sub-types of children that may have different contributing factors to neurodevelopment and, as has been highlighted recently [27, 28], opportunities for novel therapies to improve outcomes.

These findings broadly align with previous research in paediatric populations that have shown pro-inflammatory mediators, which are hallmarks of the atopic response, to correlate with the severity of behavioural outcomes in ASD [19, 29]. One plausible explanation for these findings may relate to the epithelial barrier dysfunction associated with atopic conditions [15, 19]. For example, aberrant tight junction formation in the airway, intestinal and/or cutaneous epithelium of children with atopic diseases may have eased the invasion of external irritants, inducing a hypersensitive immune response [4, 15, 30]. Given that the initial phases of this response are believed to be predominately mediated by Th_2_ cells, interleukin (IL)-4 and IL-13 are released, promoting isotypic class switching and immunoglobulin-E production in B cells [4]. Consequently, Th_1_ immuno-active cytokines and chemokines are released, and mast cells are recruited to synthesise additional prostaglandins, tumour necrosis factor and vascular endothelial growth factor [4, 30]. Interestingly, previous research indicates that, when chronic, this atopic response is associated with reduced integrity of the BBB, likely increasing cytokine entry into the brain, and facilitating cortical disruption in circuits regulating early life development, relative to non-atopic participants [16, 17]. Overall, the convergence between our findings and prior studies offers support for the notion that atopic conditions could potentially influence social outcomes in children with ASD, however further prospective, longitudinal research is needed to establish directional and causative pathways.

Alternatively, the current findings may be underpinned by a model whereby severe ASD-related symptomatology, including higher sensory sensitivity, may exacerbate cutaneous afflictions. For instance, given that children with autism regularly report hypo- and hyper-responsivity to touch, the observed difference in symptom severity between eczematic and asthmatic/allergic participants may be underpinned by ASD-related differences in sensory processing [31, 32]. In typically developing eczematic children, excessive scratching and severe pruritis have been correlated with intense irritation and seclusion [33]. It seems plausible that both cutaneous symptoms and their associated adverse behavioural outcomes may be exacerbated in children with ASD who present with severe tactile sensitivity. Results may indicate that severe sensory sensitivity may be associated with intensified cutaneous discomfort and act as a mediator of worsened behavioural outcomes in eczematic children with ASD. Future research is warranted to examine the prevalence of tactile processing difficulties amongst children with ASD and eczema relative to other atopic conditions, and whether this relates to ASD symptom severity.

Our study further explored possible alternative determinants proposed in previous literature to mediate variation in ASD-related symptom severity. For instance, prenatal exposure to pro-inflammatory mediators, such as that stemming from maternal immune activation, has been associated with an increase in the severity of emotional and behavioural impairments in children with ASD [26, 34]. The current study, however, detected no significant differences in reports of maternal infection during pregnancy between the atopic and non-atopic cohorts. Consequently, gestational immune activation was unlikely a key factor underlying the observed differences in ASD symptom severity between these groups. Similarly, prior literature has pointed to an association between sleep disorders and adverse behavioural outcomes in children with atopic conditions [4, 14, 35]. In our study, however, no significant differences in sleep pattern were determined between our atopic and non-atopic cohorts, indicating that abnormal sleep behaviours were unlikely a confounder in the observed difference in symptom severity between the two groups.

By contrast, the results of this study did not support a link between non-social symptoms, namely restricted and repetitive behaviours, and reports of atopic disease. These findings are consistent with the difficulties of previous research to detect an effect in the RRB domain, with a distinct heterogeneity in the prevalence and severity of repetitive and stereotyped actions possibly attributable to these behaviours being less naturally evoked [25]. Further, prior research has established ceiling effects in this domain for young children displaying lower-percentile language ability, which resembles the current sample [23]. Overall, our study points to the need for future research into alternative severity metrics for evaluating restricted and repetitive interests and behaviours in paediatric populations.

The present study has several limitations. Our sample was one of convenience, consisting of children attending assessment services at university and hospital clinics. Accordingly, this population may not be representative of a broader community sample of children with ASD. Second, the current study is cross-sectional. As a result of the relatively small sample size, some findings, such as the difference in ASD symptom severity between children with eczema and children with other atopic conditions, only reached statistical significance when using one-tailed tests, supporting the need for further research with larger samples. Additionally, instances of atopic diseases deviated notably from those reported in previous literature, with a possible under-estimation observed for hay fever [6, 7]. This may be due to the reliance on self-report within a medical interview for atopic disease, rather than evaluating allergen-specific serum IgE or skin prick testing, possibly enabling some misclassification of atopic and non-atopic participants. Our study further did not collect biological measures of immune activation, which would have improved our understanding of the potential biological mechanisms at play. Additionally, given the proposed role of gut microbiota and metabolites in the pathophysiology of ASD [2] and the development of potential treatments targeting gut-derived metabolites to improves symptoms associated with ASD [27], further investigations should consider the role of gastrointestinal features alongside atopic features.

Despite these limitations, the present study provides valuable insights into possible determinants and predictors of neurodevelopment and ASD symptomatology. Overall, our findings support the notion that atopic conditions, particularly cutaneous syndromes, may be related to the severity of overall and social domain outcomes in children with ASD. This study adds to the growing body of literature examining atopic disorder’s association with ASD by proposing that atopic disease may be a factor in the phenotypic expression of autism symptom severity in a subgroup of children.

## Supplementary information

Supplementary Information: Table 1
