# Supplementary material for: Eczema and related atopic diseases are associated with increased symptom severity in children with autism spectrum disorder
Source: Transl Psychiatry. 2022 Sep 28;12:415. doi: 10.1038/s41398-022-02185-5 (PMC9519885; doi:10.1038/s41398-022-02185-5)
Supplement: Supplementary file 1 — Supplementary Information: Table 1 [file 41398_2022_2185_MOESM1_ESM.docx]

Supplementary Information

**TABLE 1** Means, standard deviations, range, and assessments of normality

|  | Mean | SD | Range | Skewness; Kurtosis | Shapiro Wilk |
| --- | --- | --- | --- | --- | --- |
| Age (years) | 6.14 | 2.62 | 2.47 – 12.89 | 0.92; 0.04 | 0.91^**^ |
| ADOS-2 CSS | 7.37 | 1.77 | 3-10 | -0.32; 0.11 | 0.93^**^ |
| ADOS-2 CSS-SA | 7.20 | 1.93 | 2-10 | -0.35; -0.31 | 0.95^**^ |
| ADOS-2 CSS-RRB | 7.44 | 2.14 | 1-10 | -1.19; 1.31 | 0.87^**^ |

Abbreviations: ADOS-2, autism diagnostic observation schedule, second edition; CSS, calibrated severity scores; SA, social affect; RRB, restricted and repetitive behaviour.
Percentage missing data for full cohort: ADOS-2 all scales 3.57%.
 **p < 0.001.
